# Supplementary figures and images for: The Tyrosine Phosphatase PRL Regulates Attachment of Toxoplasma gondii to Host Cells and Is Essential for Virulence
Source: mSphere. 2022 May 23;7(3):e00052-22. doi: 10.1128/msphere.00052-22 (PMC9241511; doi:10.1128/msphere.00052-22)

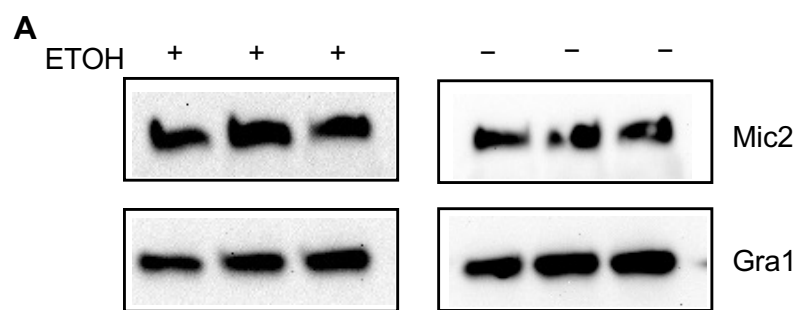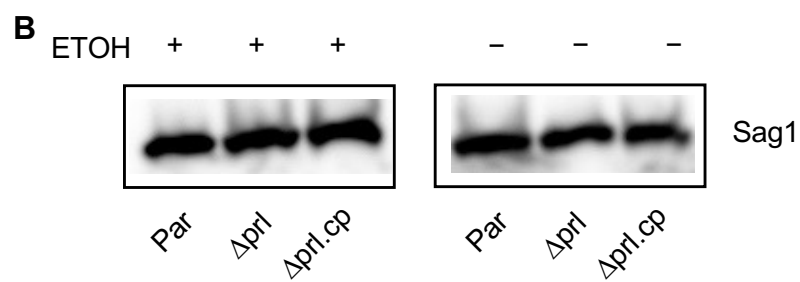

Supplement: FIG S1 [file msphere.00052-22-sf001.pdf]

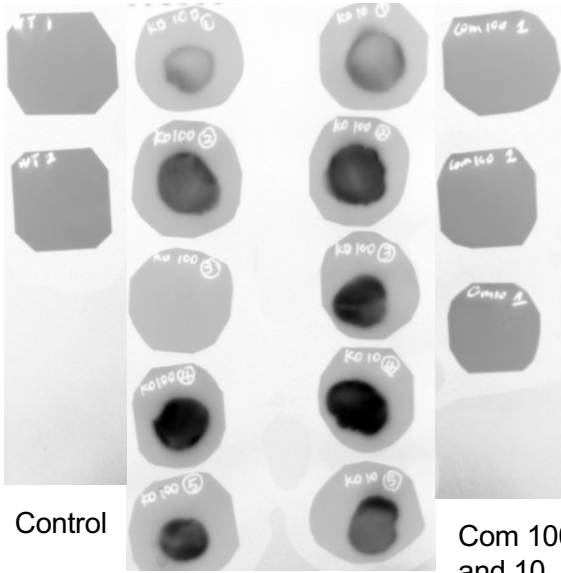

Control

KO 100

KO 10

Com 100  
and 10

Supplement: FIG S2 [file msphere.00052-22-sf002.pdf]
