# Supplementary material for: The Tyrosine Phosphatase PRL Regulates Attachment of Toxoplasma gondii to Host Cells and Is Essential for Virulence
Source: mSphere. 2022 May 23;7(3):e00052-22. doi: 10.1128/msphere.00052-22 (PMC9241511; doi:10.1128/msphere.00052-22)
Supplement: DATA SET S1 [file msphere.00052-22-sd001.pdf]

ID	Annotation	Fold Change	Control (Δku80)	Δprl.cp
TGGT1_208718	putative protein tyrosine phosphatase type IVA A	INF	0	54
TGGT1_287980	FHA domain-containing protein	INF	0	17
TGGT1_202840	FHA domain-containing protein	INF	0	12
TGGT1_262150	kelch repeat and K+ channel tetramerisation domain containin	INF	0	11
TGGT1_278660	putative P-type ATPase4	INF	0	9
TGGT1_211350	CBS domain-containing protein	INF	0	8
TGGT1_297520	proteophosphoglycan PPG1	INF	0	8
TGGT1_219700	putative DNA replication licensing factor MCM4	INF	0	6
TGGT1_227800	EF hand domain-containing protein	INF	0	6
TGGT1_243920	putative DNA replication licensing factor MCM5	INF	0	6
TGGT1_263130	putative citrate synthase	INF	0	6
TGGT1_289970	hypothetical protein	INF	0	5
TGGT1_311830	hypothetical protein	INF	0	5
TGGT1_204310	hypothetical protein	INF	0	4
TGGT1_229650	josephin protein	INF	0	4
TGGT1_235490	hypothetical protein	INF	0	4
TGGT1_242800	putative ribosome biogenesis protein NSA2	INF	0	4
TGGT1_300060	signal peptidase subunit protein	INF	0	4
TGGT1_208590	putative vacuolar ATP synthase subunit 54kD	INF	0	3
TGGT1_214970	putative DNA replication licensing factor	INF	0	3
TGGT1_224130	hypothetical protein	INF	0	3
TGGT1_238040A	protein disulfide-isomerase domain-containing protein	INF	0	3
TGGT1_252430	hypothetical protein	INF	0	3
TGGT1_253440	putative cell-cycle-associated protein kinase SRPK	INF	0	3
TGGT1_255320	mRNA turnover 4 (MRT4) family protein	INF	0	3
TGGT1_262710	Ctr copper transporter family protein	INF	0	3
TGGT1_272600	adaptin c-terminal domain-containing protein	INF	0	3
TGGT1_293860	hypothetical protein	INF	0	3
TGGT1_294640	ribonucleoside-diphosphate reductase large chain	INF	0	3
TGGT1_297940	single-strand binding protein	INF	0	3
TGGT1_299780	hypothetical protein	INF	0	3
TGGT1_203370	hypothetical protein	INF	0	2
TGGT1_209210	hypothetical protein	INF	0	2
TGGT1_213770	putative Superkiller viralicidic activity 2 family 2	INF	0	2
TGGT1_214180	ENTH domain-containing protein	INF	0	2
TGGT1_216730	MCM2/3/5 family protein	INF	0	2
TGGT1_216810	5'-nucleotidase, C-terminal domain-containing protein	INF	0	2
TGGT1_223680	ubiquitin family protein	INF	0	2
TGGT1_226580	hypothetical protein	INF	0	2
TGGT1_227650	putative microtubule-associated protein RP/EB family	INF	0	2
TGGT1_227970	histone family DNA-binding protein	INF	0	2
TGGT1_233000	KOW motif domain-containing protein	INF	0	2
TGGT1_237220	putative DNA replication licensing factor Mcm7	INF	0	2
TGGT1_239790	BRCA1 C Terminus (BRCT) domain-containing protein	INF	0	2
TGGT1_245470	mitotic checkpoint protein, BUB3 family protein	INF	0	2
TGGT1_245490	microneme protein MIC8	INF	0	2
TGGT1_248890	actin-like protein ALP3b	INF	0	2
TGGT1_249610	hypothetical protein	INF	0	2
TGGT1_259260	membrane protein FtsH1	INF	0	2
TGGT1_261000	MutS domain V domain-containing protein	INF	0	2
TGGT1_263750	hypothetical protein	INF	0	2
TGGT1_264770	hypothetical protein	INF	0	2
TGGT1_269180	MIF4G domain-containing protein	INF	0	2
TGGT1_270780	DNA-directed RNA polymerase II RPB6	INF	0	2
TGGT1_271970	glideosome-associated protein with multiple-membrane spans	INF	0	2

TGGT1_285250	hypothetical protein	INF	0	2
TGGT1_286160B	non-specific serine/threonine protein kinase	INF	0	2
TGGT1_286240	putative kelch repeat protein	INF	0	2
TGGT1_292120	membrane occupation and recognition nexus protein MORN2	INF	0	2
TGGT1_297130	hypothetical protein	INF	0	2
TGGT1_299810	cysteine-tRNA synthetase (CysRS)	INF	0	2
TGGT1_306210	RNA polymerase II accessory factor CDC73	INF	0	2
TGGT1_307570	putative glycerol-3-phosphate dehydrogenase (gpdh)	INF	0	2
TGGT1_308810B	rhoptry neck protein RON9	INF	0	2
TGGT1_310060	small nuclease	INF	0	2
TGGT1_310360	hypothetical protein	INF	0	2
TGGT1_310440	membrane occupation and recognition nexus protein MORN1	INF	0	2
TGGT1_311030	hypothetical protein	INF	0	2
TGGT1_313240	putative ethylene-responsive RNA helicase	INF	0	2
TGGT1_314400	putative pyruvate dehydrogenase E1 component, beta subunit	10	1	10
TGGT1_219860	putative replication licensing factor	9	1	9
TGGT1_227560	putative IWS1 transcription factor	9	1	9
TGGT1_253940	CAM Kinase family, incomplete catalytic triad	9	1	9
TGGT1_305510	hypothetical protein	9	1	9
TGGT1_311625	WD domain, G-beta repeat-containing protein	9	1	9
TGGT1_308860	hypothetical protein	7	1	7
TGGT1_211690	AAA family protein	6	1	6
TGGT1_227948	peptidase M16 inactive domain-containing protein	6	1	6
TGGT1_231210	sarcalumenin/eps15 family protein	6	1	6
TGGT1_202530	aspartate-tRNA ligase	5	1	5
TGGT1_205010	putative U2 small nuclear ribonucleoprotein family protein	5	1	5
TGGT1_253900	parasite porphobilinogen synthase PBGS	5	1	5
TGGT1_260630	DnaJ family Sec63 protein	5	1	5
TGGT1_277910	thrombospondin type 1 domain-containing protein	5	1	5
TGGT1_320210	WD domain, G-beta repeat domain containing protein	5	1	5
TGGT1_271050	SAG-related sequence SRS34A (SAG2A)	4	3	12
TGGT1_212090	hypothetical protein	4	2	8
TGGT1_206610	pyruvate dehydrogenase complex subunit PDH-E2	4	1	4
TGGT1_214210	rRNA pseudouridine synthase	4	1	4
TGGT1_218240	hypothetical protein	4	1	4
TGGT1_225125	pre-rRNA processing protein	4	1	4
TGGT1_240850	WD domain, G-beta repeat-containing protein	4	1	4
TGGT1_245670	pyruvate dehydrogenase complex subunit PDH-E1Alpha	4	1	4
TGGT1_272290	pyruvate dehydrogenase complex subunit PD-HE1Beta	4	1	4
TGGT1_298610	GYF domain-containing protein	4	1	4
TGGT1_309740	LSM domain-containing protein	4	1	4
TGGT1_314410	putative aquarius	4	1	4
TGGT1_315690	DnaJ domain-containing protein	4	1	4
TGGT1_243960	nuclear transport factor 2 (ntf2) domain-containing protein	3.5	2	7
TGGT1_251790	hypothetical protein	3.5	2	7
TGGT1_270550	putative gamma-glutamyl phosphate reductase	3.5	2	7
TGGT1_222220	alveolin domain containing intermediate filament IMC7	3	4	12
TGGT1_212250	XPG N-terminal domain-containing protein	3	2	6
TGGT1_226050	hypothetical protein	3	2	6
TGGT1_233110	IMP dehydrogenasa (IMPDH)	3	2	6
TGGT1_261440	ARM repeats containing protein	3	2	6
TGGT1_297470	putative myosin light chain 2	3	2	6
TGGT1_202610	protein phosphatase 2C domain-containing protein	3	1	3
TGGT1_204490	hypothetical protein	3	1	3
TGGT1_229930	p25-alpha family protein	3	1	3
TGGT1_230350	hypothetical protein	3	1	3

TGGT1_231130	hypothetical protein	3	1	3
TGGT1_232830	putative vacuolar proton translocating ATPase subunit A	3	1	3
TGGT1_244150	glycerate kinase	3	1	3
TGGT1_252630	hypothetical protein	3	1	3
TGGT1_253730	importin-beta N-terminal domain-containing protein	3	1	3
TGGT1_254390	CRAL/TRIO domain-containing protein	3	1	3
TGGT1_257490	prefoldin subunit superfamily protein	3	1	3
TGGT1_266970	hypothetical protein	3	1	3
TGGT1_268950	hypothetical protein	3	1	3
TGGT1_269650	FFD and TFG box motifs protein	3	1	3
TGGT1_277760	putative adenylosuccinate lyase	3	1	3
TGGT1_285850	peptidyl-prolyl cis-trans isomerase, FKBP-type domain-contain	3	1	3
TGGT1_288350	hypothetical protein	3	1	3
TGGT1_289600	heat shock protein HSP29	3	1	3
TGGT1_300120	aminotransferase, class V superfamily protein	3	1	3
TGGT1_305980	pyruvate dehydrogenase complex subunit PDH-E3I	3	1	3
TGGT1_306330	phospholipase	3	1	3
TGGT1_309265	oxidoreductase, short chain dehydrogenase/reductase family p	3	1	3
TGGT1_310760	protein phosphatase 2C domain-containing protein	3	1	3
TGGT1_311360	protein kinase G AGC kinase family member PKG	3	1	3
TGGT1_311480	hypothetical protein	3	1	3
TGGT1_313640	hypothetical protein	3	1	3
TGGT1_314080	hypothetical protein	3	1	3
TGGT1_316900	Sas10 C-terminal domain-containing protein	3	1	3
