# Supplementary material for: The Tyrosine Phosphatase PRL Regulates Attachment of Toxoplasma gondii to Host Cells and Is Essential for Virulence
Source: mSphere. 2022 May 23;7(3):e00052-22. doi: 10.1128/msphere.00052-22 (PMC9241511; doi:10.1128/msphere.00052-22)
Supplement: TABLE S1 [file msphere.00052-22-st001.pdf]

Purpose	Name	Sequence
Amplify the promotor of TgPRL gene	prl.pr.F1	GCGAATTGGGTACCGGGCCCTTTAGTGACGCCAGAAACGTAAG
	prl.pr.R1	TACGGGTACATCTTTCTAGCGAAAAAGTGAG
Amplify the CDS of TgPRL gene with an HA tag in the N-terminal	prl.cds.F1	AAAGATGTACCCGTACGACGTCCCGGACTACGCTAACGAAGCGTCGTCCTGC
	prl.cds.R1	TttatctagacttaagTCACATGATTGCGCATCTACCTCCG
Generate pSag1-Cas9-U6-sgPRL by using Q5 mutagenesis	prl.ko.sgRNA.F1	gtgtagactgGTTTTAGAGCTAGAAATAGC
	prl.ko.sgRNA.R1	tgctcccttcAACTTGACATCCCCATTTAC
Amplify the DHFR selection cassette from the plasmid pJET-DHFR	prl.ko.hr.dhfr.F1	GTTGGGCCACAGCCCGACGACCAGCCACTCGGAACTGACCAGCGACTCACTATAGGGAGAGCGGC
	prl.ko.hr.dhfr.R1	CGCTCCTCTTCTCCTCTGTCTTCTCTTCGTTTCCTCCGCGGCAAGAACATCGATTTTCCATGGCAG
Test Δprl shown in Figure 2	P1.F	TTTTGACGCGTCTCTGTAGGTA
	P1.R	atgtggcatttcacacagtctc
	P2.F	gaggagagacggaaagtgctta
	P2.R	AATCTGGCTGTCTAAAGCCTTG
Generate pSag1-Cas9-U6-sgKU80 by using Q5 mutagenesis	sgKU80.F1	ctcatattccGTTTTAGAGCTAGAAATAGC
	sgKU80.R1	aaaggtgtacAACTTGACATCCCCATTTAC
Amplify the HA-PRL expression cassette with short homology to the 5' UTR of ku80 gene	PRL.ku80.EE.HR.F2	GTCCCCGGTTCGCCTCAGCACACACACACATGACGTACATCGcgcggcttatttagttaagggagc
	PRL.ku80.EE.HR.R1	GTAATGTCGGAATAGTTCCCATCAGAAACAATGGAGCTATCCCAAAAGCTGGAGCTCGTACC

Artificially synthesized fragment for generation of C339S mutant	C339S-synthesis-DNA	GTGTAGACTGCGGCCGCCGCGGAGGAAACGAAGAGA<br>AGACAGAGGAGAAGAGGAGCGAGAAGGCTGGGGCCG<br>AGCGCGAGTGCGAGAGGAAGACGCAGCGACCTCGAT<br>TTGACGGCGACGGTTCTTCTCTCAGCCCGCGAAGCAA<br>CGCGTCCCGCGGCAGCGCGTGCAGCACACGGAGCTG<br>CGAAAGCCACGAGAAGGTTGGCCGCACGTTGAGTGG<br>AGACTCGACGGGCAGAGACGGCGTCTGTTCTGTCTCT<br>TCTGCTCGCCAGCCTTCTCTCAGGAGAAAAAGCGCCG<br>CTGAGGAAGTGGGACGCGAGGTGAAGGTCGACGGCA<br>AGGCTTTAGACAGCCAGATTGAACTTGTGCGGACGAA<br>GAACAGGGGCAAGAGCGAGGTGACCCGACTGGGTCG<br>CGGGGCGGGGAGTGCATGCGCGGTGTCAGTGTCGCC<br>TCTCGGGACTGCGCGTCACGTTCCTTCCGGGCGCCAA<br>CCGAGTGAAAGGCGCCAGAGCGCGAAGCCTCTGACG<br>AAGCTCAGTTCGTCGTCCTCCAGCCACAACAAGACAA<br>CGATGGTGCCGACTGTGATGAACACGCCGACCCGCAT<br>CGAGGCGGGGCGTCAGAAGTTTCTGATTTTCGACGCG<br>CCTAGCCAGGAGAATCTTCCCGCGTACATCGAGGAAA<br>TGCGCGCCTACGAGGTCACAGACTTGGTGCGAACCTG<br>CGAGCGGACCTACGACGACAAGACCGTGCTGGCTTCC<br>GGCATCCGCCCCCACGAACTGATCTTCCCCGATGGAG<br>AGGCGCCTCCAGACGATGTCATCGACGAGTGGCTCAC<br>GCTCTGCAACGCCGTCTCGCAGCAGAGGGGCGCAAT<br>CGCGATTCACAGCGTCGCTGGTTTAGGTCGCGCCCCG<br>GTCCTCGTCGCCATCGCCCTCATCGAAAAGGGCATGG<br>ACCCCATGGATGCCATCAT
Artificially synthesized fragment for generation of C401A mutant	C401A-synthesis-DNA	GGCATGGACCCCATGGATGCCATCATGTTCATTCGAG<br>AAAGAAGAAAGGGCGCCATCAACCGA
